# Prolyl hydroxylase 2 inactivation enhances glycogen storage and promotes excessive neutrophilic responses

**DOI:** 10.1172/JCI90848

**Published:** 2017-08-14

**Authors:** Pranvera Sadiku, Joseph A. Willson, Rebecca S. Dickinson, Fiona Murphy, Alison J. Harris, Amy Lewis, David Sammut, Ananda S. Mirchandani, Eilise Ryan, Emily R. Watts, A.A. Roger Thompson, Helen M. Marriott, David H. Dockrell, Cormac T. Taylor, Martin Schneider, Patrick H. Maxwell, Edwin R. Chilvers, Massimilliano Mazzone, Veronica Moral, Chris W. Pugh, Peter J. Ratcliffe, Christopher J. Schofield, Bart Ghesquiere, Peter Carmeliet, Moira K.B. Whyte, Sarah R. Walmsley

**Affiliations:** 1 MRC/University of Edinburgh Centre for Inflammation Research, Queen’s Medical Research Institute, University of Edinburgh, Edinburgh, United Kingdom.; 2 Laboratory of Angiogenesis and Vascular Metabolism, Vesalius Research Center, Leuven, Belgium.; 3 Academic Unit of Respiratory Medicine and; 4 Academic Unit of Immunology and Infectious Diseases, Department of Infection, Immunity and Cardiovascular Disease, The Medical School, University of Sheffield, Sheffield, United Kingdom.; 5 UCD School of Medicine and Medical Science, Conway Institute, University College Dublin, Dublin, Ireland.; 6 General, Visceral and Transplantation Surgery, University of Heidelberg, Heidelberg, Germany.; 7 Department of Medicine, University of Cambridge, Cambridge, United Kingdom.; 8 Laboratory of Tumour Inflammation and Angiogenesis, Department of Oncology, Leuven, Belgium.; 9 Nuffield Department of Medicine and; 10 The Department of Chemistry, University of Oxford, Oxford, United Kingdom.

**Keywords:** Inflammation, Metabolism, Innate immunity, Neutrophils, hypoxia

## Abstract

Fully activated innate immune cells are required for effective responses to infection, but their prompt deactivation and removal are essential for limiting tissue damage. Here, we have identified a critical role for the prolyl hydroxylase enzyme *Phd2* in maintaining the balance between appropriate, predominantly neutrophil-mediated pathogen clearance and resolution of the innate immune response. We demonstrate that myeloid-specific loss of *Phd2* resulted in an exaggerated inflammatory response to *Streptococcus pneumonia*, with increases in neutrophil motility, functional capacity, and survival. These enhanced neutrophil responses were dependent upon increases in glycolytic flux and glycogen stores. Systemic administration of a HIF–prolyl hydroxylase inhibitor replicated the *Phd2*-deficient phenotype of delayed inflammation resolution. Together, these data identify *Phd2* as the dominant HIF-hydroxylase in neutrophils under normoxic conditions and link intrinsic regulation of glycolysis and glycogen stores to the resolution of neutrophil-mediated inflammatory responses. These results demonstrate the therapeutic potential of targeting metabolic pathways in the treatment of inflammatory disease.

## Introduction

Inappropriate or persistent neutrophilic inflammation is implicated in a number of disease states exemplified by acute lung injury responses and chronic obstructive pulmonary disease. There are, to date, no effective therapeutic strategies for targeting neutrophilic inflammation, a reflection in part of the fine balance that exists between maintaining effective host pathogen responses and limiting host-mediated tissue damage. Local and systemic hypoxia represent a critical component of the inflammatory response, with oxygen-sensing pathways implicated in the regulation of neutrophil survival. The coordination of cellular responses to differing oxygen tensions is tightly controlled and involves hydroxylation of the HIF subunits, with prolyl hydroxylation targeting HIFs for ubiquitylation and proteosomal degradation (1, 2). In humans, 3 prolyl hydroxylase enzymes are described (PHD1–3), with PHD2 the most abundant isoform and important in setting basal levels of HIF-1α in the majority of cells cultured in normoxia (3–6). While there is emerging evidence that genetically observed PHD2 variations result in distinct clinical phenotypes (7–9), the overall physiological roles of PHD2 are only now emerging. An important example is the critical role of PHD2 in macrophage polarization, with haplodeficiency of *Phd2* resulting in expansion of tissue-resident M2 macrophages with consequences for arteriogenesis (10).

We have previously observed that neutrophils express all 3 PHDs. PHD2 is both expressed constitutively and upregulated in sterile inflammation, where PHD2 expression follows HIF activity. In marked contrast to PHD3 expression, however, only modest changes in *PHD2* transcript are observed in response to hypoxic cell culture (11). HIF-1α, HIF-2α, and PHD3 have all been shown to regulate neutrophil survival and function, with implications for neutrophilic inflammation (11–13). In vivo inactivation of both *Hif1α* and *Phd3* results in abolished hypoxic survival and loss of functional capacity. Inactivation of *Hif2α* is also associated with a reduction in neutrophilic inflammation and tissue damage, as a consequence of increased neutrophil apoptosis. This suggests PHD2 is likely to also play a role in mechanisms regulating neutrophil apoptosis. Conceptually, it is also likely that individual PHD family members play distinct roles in regulating key neutrophil functional and survival responses under differing oxygen tensions (11, 12, 14).

An increasing body of work has focused on the potential roles of prolyl hydroxylases as metabolic sensors, in addition to their role as oxygen sensors, both indirectly through their regulation of HIF-mediated signaling and directly through their dependence on the tricarboxylic acid (TCA) cycle intermediate α-ketoglutarate (α-KG) and their ability to interact with the glycolytic enzyme pharmacokinetics (PK) (1, 15–20). This linking of metabolism to cell survival and function has important implications for the role of PHD enzymes in regulating myeloid cell functional responses, particularly in light of recent macrophage studies linking metabolic status to macrophage polarization and immune signaling (21–23). In neutrophils, we observed that mutations in succinate dehydrogenase B result in enhanced neutrophil survival responses, despite evidence of a dysfunctional TCA cycle (24), directly linking metabolism with neutrophil survival. The importance of changes in the metabolic capacity of neutrophils and, more specifically, flux between individual metabolic pathways under resting and activated states remains to be fully explored, as does the role PHD enzymes may play in coordinating these responses.

In this work, we address the consequences of myeloid-specific loss of *Phd2* for host-pathogen interactions and link the metabolic state of neutrophils to survival and functional status. Our findings reveal that loss of *Phd2* results in increases in neutrophil activation and persistence, resulting in increased tissue injury following challenge with intratracheal *Streptococcus pneumonia*. We demonstrate that enhanced survival and function of *Phd2*-deficient neutrophils is directly linked to their metabolic capacity and that modulating neutrophil metabolism can alter outcomes of infection and inflammation.

## Results

### Myeloid-specific deletion of Phd2 results in enhanced neutrophilic inflammation and lung injury.

**Figure 1:**
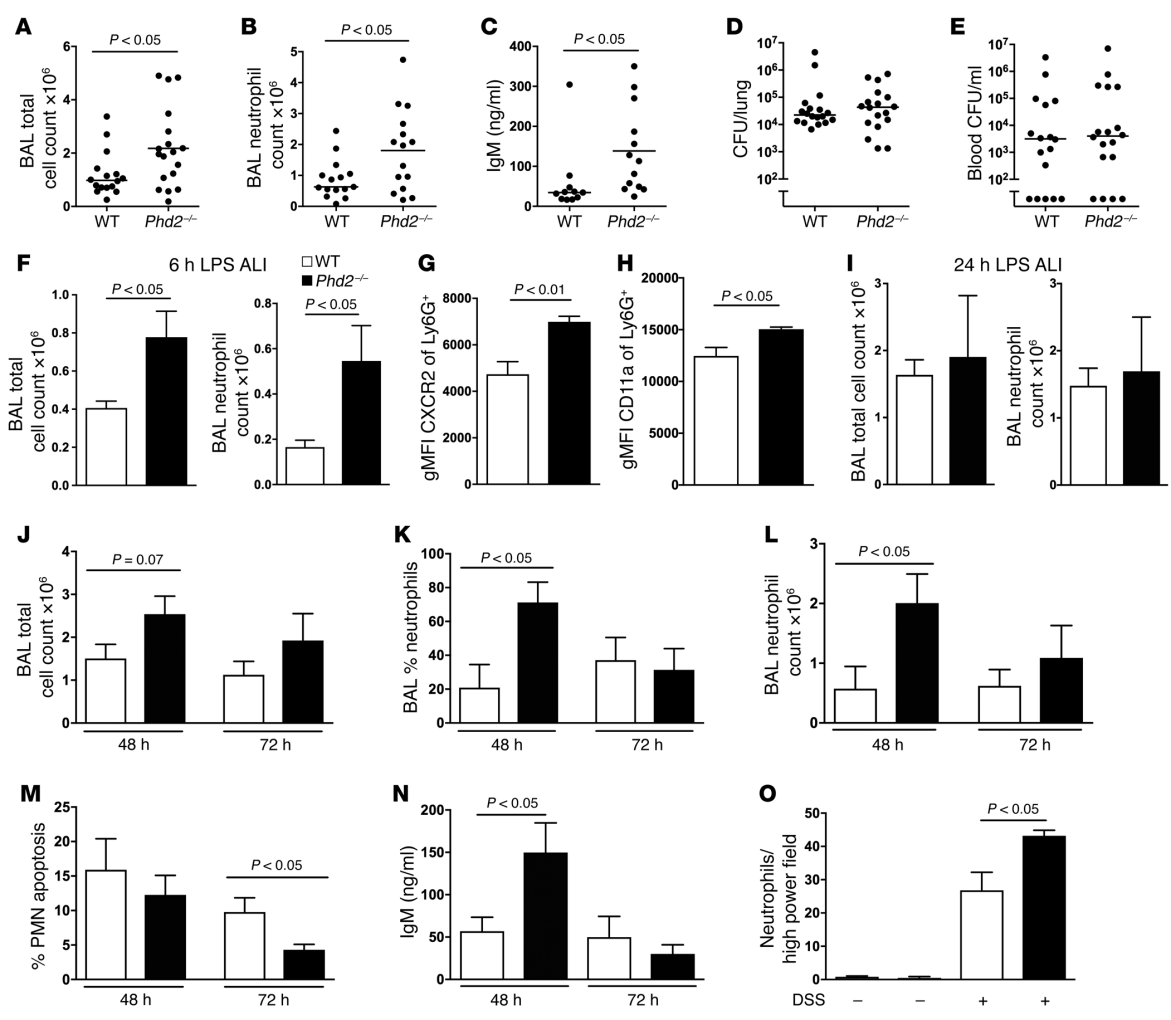
Myeloid-specific *Phd2* deficiency results in aberrant neutrophilic inflammation. WT (white bars) and myeloid-specific *Phd2^–/–^* (black bars) mice were studied in parallel. (**A**–**E**) Mice were infected via the trachea with 1 × 10^7^ CFU of TIGR4. Cells were harvested by BAL at 14 hours and total cell counts (**A**) and neutrophil differential counts (**B**) obtained. Total IgM release into bronchoalveolar fluid (**C**) and viable bacterial counts recovered from homogenized lung (**D**) or whole blood (**E**) were performed in parallel (*n* = 15). (**F**–**L**) Acute lung injury. Intratracheal LPS (0.3 mg) was instilled in anesthetized mice. Mice were sacrificed at 6 hours after challenge and cells harvested by BAL for total cell counts (**F**) and surface expression of CXCR2 (**G**) and CDlla (**H**). At 24 (**I**), 48, and 72 hours (**J**–**N**) after challenge, cells/supernatants were harvested by BAL for total cell counts (**I**, **J**, **L**), neutrophil differential counts (**I**, **K**, **L**), morphological counts of apoptosis (**M**), and measures of total IgM release (**N**) (*n* = 7). (**O**) DSS colitis. Six days following DSS diet–induced colitis, colonic sections were harvested, paraffin-fixed, and anti-MPO antibody stained. ALI, acute lung injury. *P* values obtained via unpaired *t* test.

To investigate the consequences of *Phd2* loss for host pathogen responses, we challenged *Phd2^fl/fl^ LysM-Cre* positive and negative (hereafter myeloid-specific *Phd2^–/–^* and WT, respectively) littermate controls with serotype 4 *S*. *pneumoniae* (TIGR4) in a model of fulminant bacterial pneumonia with associated secondary bacteremia. Loss of *Phd2*, verified in neutrophil and bone marrow–derived macrophage (BMDM) populations by real-time PCR (Supplemental Figure 1, A and B; supplemental material available online with this article; https://doi.org/10.1172/JCI90848DS1), resulted in higher total (Figure 1A) and neutrophil (Figure 1B) bronchoalveolar lavage (BAL) cell counts, with a corresponding increase in levels of BAL IgM, a marker of vascular injury (Figure 1C). The bacterial burden was unchanged both regionally in the lung (Figure 1D) and in the circulation (Figure 1E). Given the augmented inflammatory response observed following challenge with a live bacterial pathogen, in which both early macrophage and subsequent neutrophil responses are required for effective pathogen clearance (25), we proceeded to define the importance of myeloid-specific expression of *Phd2* in another murine model of acute lung injury that is more specifically mediated by neutrophils. Following intratracheal challenge of mice with bacterial lipopolysaccharide (LPS) during the early phase of neutrophil recruitment (6 hours after LPS), myeloid-specific *Phd2^–/–^* mice displayed increased lavage total cell counts, largely reflecting increased neutrophil numbers (Figure 1F). This was associated with a normal *Phd2^–/–^* baseline (Supplemental Figure 1C) and LPS-stimulated total white cell and differential cell counts (Supplemental Figure 1D) in blood and correlated with enhanced levels of the neutrophil chemokines KC and MIP-1B (Supplemental Figure 2, A–D). In keeping with a neutrophil-intrinsic response, *Phd2*-deficient airway neutrophils expressed higher levels of CXCR2 and CD11a (Figure 1, G and H), with no effect of *Phd2* loss on BMDM cytokine expression (Supplemental Figure 2, E–H), as previously reported (26). Importantly, maximal neutrophil recruitment reached equivalence between genotypes at 24 hours, suggesting *Phd2^–/–^* mice had more rapid neutrophil recruitment rather than recruiting greater cell numbers (Figure 1I). In keeping with the phenotype observed following pulmonary infection with *S*. *pneumoniae*, we found delayed inflammation resolution (Figure 1J) and neutrophil persistence (Figure 1, K and L) in *Phd2^–/–^* animals and a significant reduction in detectable neutrophil apoptosis (Figure 1M), with a parallel increase in BAL IgM release (Figure 1N). In a model of more chronic inflammation, dextran sulfate sodium (DSS) diet–induced colitis, greater neutrophil persistence was seen in heterozygous *Phd2^+/–^* than in WT mice (Figure 1O). Taken together, these data implicate *Phd2* in both the recruitment and resolution of neutrophil-mediated inflammatory responses, with potentially important consequences for resultant tissue damage.

### Phd2-deficient neutrophils display enhanced function and survival ex vivo.

**Figure 2:**
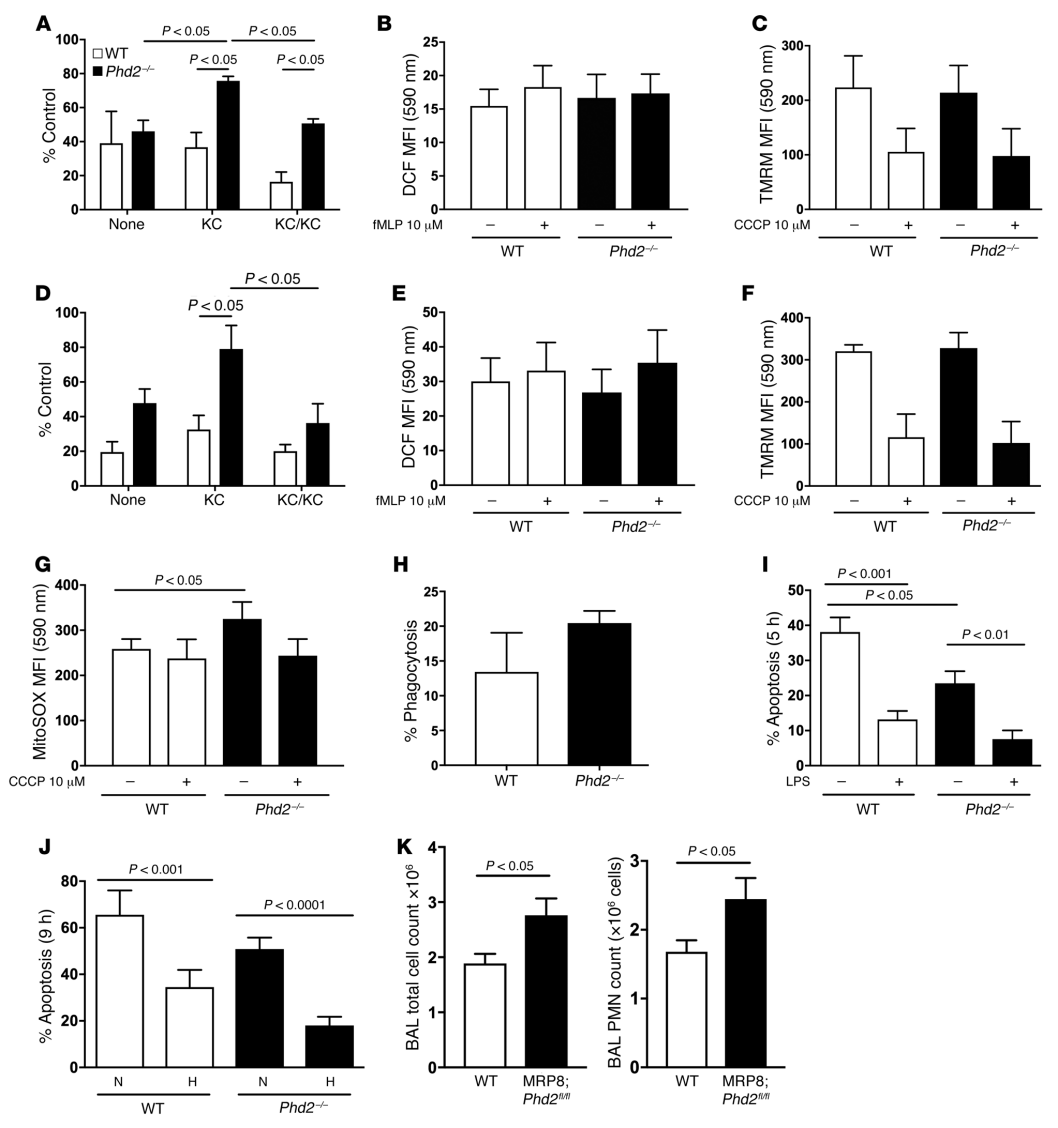
*Phd2*-deficient neutrophils display augmented inflammatory responses. (**A**–**G**) Murine inflammatory BAL neutrophils were isolated from WT (white bars) and myeloid-specific *Phd2^–/–^* (black bars) mice 24 hours after in vivo LPS (3 mg) challenge and studied either at point of isolation (**A**–**C**) or following aging in culture for 6 hours (**D**–**G**). (**A** and **D**) Chemotaxis. Chemotaxis to KC (100 nM) was determined using neuroprobe chambers. (**B** and **E**). Respiratory burst. Change in DCF emission, an indicator of cellular oxidative stress, was quantified by flow following 30 minutes stimulation with fMLP (10 μM). (**C** and **F**) Inner mitochondrial membrane potential. FL2 geometric mean fluorescence with tetramethylrhodamine, methyl ester (TMRM) uptake was quantified by flow cytometry in the presence or absence of CCCP (10 μM). CCCP was used as a negative control for the TMRM measure of inner mitochondrial membrane potential and works by collapsing the proton gradient. TMRM is a cell-permeant cationic fluorescent dye that accumulates in active mitochondria and is distributed throughout the cytosol when mitochondrial membrane potential collapses. (**G**) Mitochondrial ROS. MitoSOX fluorescent emission was quantified by flow cytometry in the presence or absence of CCCP (10 μM). (**H**) Inflammatory BAL neutrophil bacterial phagocytosis. Alexa Fluor 488–conjugated *E*. *coli* BioParticles (MOI 5:1) were administered to inflammatory BAL neutrophil for 1 hour. (**I** and **J**) Apoptosis. Peripheral blood neutrophils were isolated from WT (white bars) and knockout *Phd2^–/–^* (black bars) mice and cultured in the presence/absence of LPS (100 ng/ml) in normoxia (19 kPa, 21% O_2_) or hypoxia (3 kPa, 3% O_2_) for 5 (**I**) or 9 (**J**) hours and apoptosis assessed by morphology. (**K**) *Phd2^fl/fl^ MRP8-Cre^+/–^* (MRP8;Phd2^fl/fl^) and WT control mice were nebulized with LPS (3 mg) and cells harvested by BAL for total cell counts and neutrophil differential counts. Data represent mean ± SEM, *n* = 4. MFI, mean fluorescence intensity. *P* values obtained via 2-way ANOVA (**A**, **D**, **I**, **J**) and unpaired *t* test (**G** and **K**).

The importance of *Phd2* expression in regulating key inflammatory neutrophil functions was investigated ex vivo in neutrophils recruited to the airways at 24 hours following in vivo challenge with LPS. Freshly isolated BAL neutrophils lacking *Phd2* displayed increased basal motility in the chemokinesis control (Kc/Kc) and enhanced chemotaxis to the murine IL-8 homologue KC at the point of isolation (Figure 2A). Ex vivo stimulation with the N-formylated bacterial chemotactic peptide *N*-formylmethionyl-leucyl-phenylalanine (fMLP) was unable to induce an increase in respiratory burst activity in either genotype (Figure 2B), and no difference in inner mitochondrial membrane potential was seen (Figure 2C). Following aging for an additional 6 hours, to investigate the functional reserve capacity of the inflammatory cells, the differences in chemotactic behavior were measured and were even more apparent (Figure 2D), while respiratory burst (Figure 2E) and inner mitochondrial membrane potential (Figure 2F) remained unchanged, despite a detectable increase in mitochondrial ROS production measured by MitoSOX (Figure 2G). In keeping with the equivalent lung CFU counts observed in vivo (Figure 1D) and in contrast with BMDMs (Supplemental Figure 3A), *Phd2*-deficient neutrophils demonstrated equivalent bacterial phagocytic capacity ex vivo (Figure 2H). At both an early (Figure 2I) and late (Figure 2J) time point, peripheral blood neutrophils from *Phd2^–/–^* animals displayed reduced constitutive rates of apoptosis and preserved survival responses to both LPS (Figure 2I) and hypoxic culture (WT 34.5% ± 7.39%, *Phd2^–/–^* 18.0% ± 3.74% apoptotic) (Figure 2J), in keeping with the observed differences in apoptosis rates in vivo (Figure 1L). There was no effect of *Phd2* loss on macrophage efferocytosis (Supplemental Figure 3B). Thus, neutrophils show both increased activation and increased longevity in the absence of *Phd2*. The further augmentation of *Phd2*-deficient neutrophil survival by hypoxia implies both PHD2-dependent and -independent regulation of neutrophil apoptosis responses. To address the neutrophil specificity of the phenotype in vivo, we challenged *Phd2^fl/fl^ LysM-Cre* positive and negative mice with LPS and with serotype 2 (D39) *S*. *pneumoniae* following alveolar macrophage depletion with clodronate (Supplemental Figure 3C). In response to LPS, *Phd2*-deficient animals again demonstrated elevated 6-hour airway neutrophil counts (Supplemental Figure 3, D and E), equivalent maximal neutrophil recruitment (Supplemental Figure 3, F and G), and enhanced persistence (Supplemental Figure 3, H and I). Following *S*. *pneumoniae* challenge, there were again increased airway leukocyte counts (Supplemental Figure 3, J and K), despite equivalent bacterial burden (Supplemental Figure 3L). To more directly address the consequence of neutrophil deletion of *Phd2*, we crossed *Phd2^fl/fl^* animals with the previously reported neutrophil-specific Cre driver MRP8 (27) (Supplemental Figure 3, M and N). *Phd2^fl/fl^ MRP8-Cre^+/–^* mice displayed increased total BAL cell counts and BAL neutrophil numbers when compared with WT mice at 48 hours after LPS challenge (Figure 2K).

### Inflammatory neutrophils deficient in Phd2 display enhanced glycolytic capacity with parallel increases in both ATP and intracellular glycogen stores.

**Figure 3:**
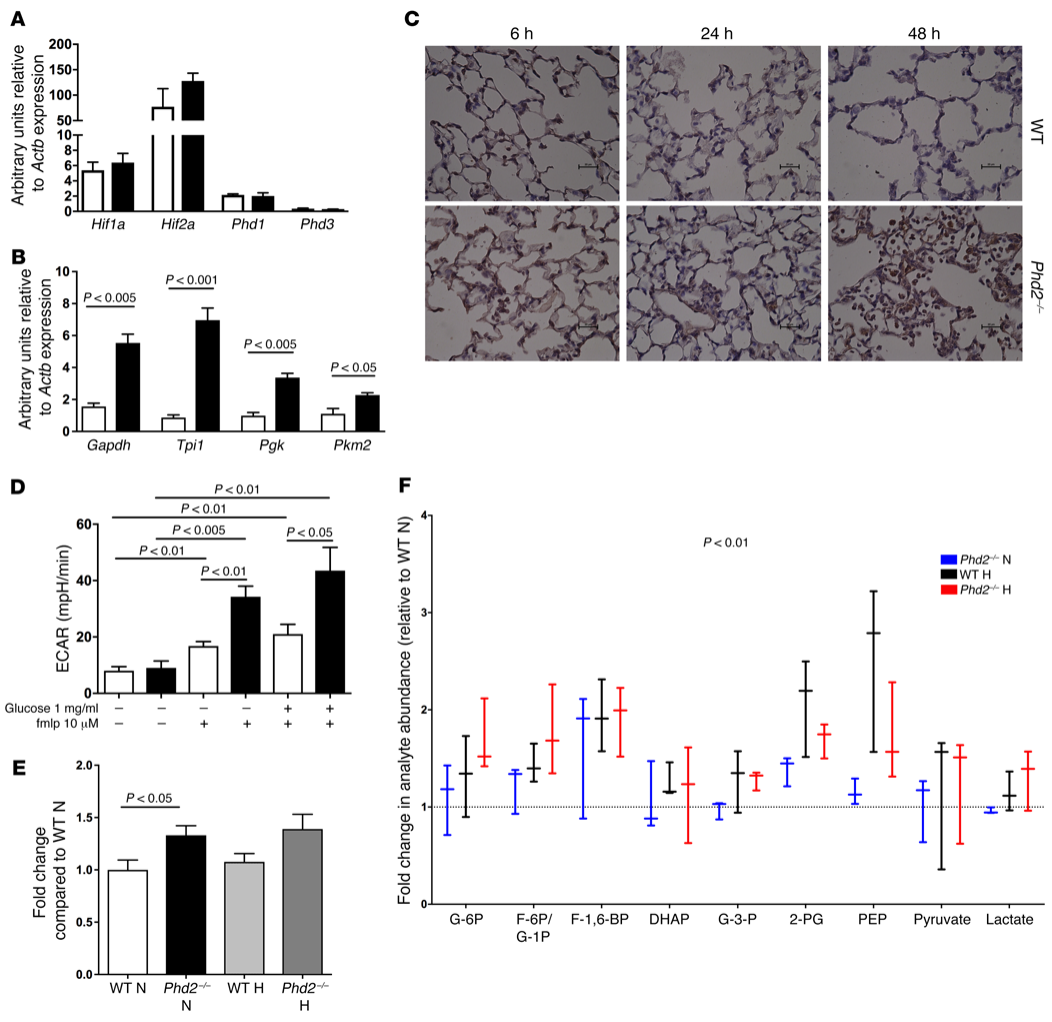
*Phd2*-deficient inflammatory neutrophils display increased HIF-1α expression and activity and enhanced glycolytic capacity. (**A** and **B**) Murine inflammatory BAL neutrophils were studied following isolation 24 hours after in vivo challenge with nebulized LPS (3 mg). WT (white bars) and myeloid-specific *Phd2^–/–^* (black bars) neutrophils were lysed and TaqMan analysis of cDNA performed with data normalized to β-actin expression. (**C**) Acute lung injury. Mice were challenged with nebulized LPS (3 mg) and sacrificed at 6, 24, and 48 hours. Lungs were fixed with 10% buffered formalin and paraffin embedded. Sections were stained for expression of HIF-1α. Original magnification, ×400. (**D**–**F**) Glycolytic capacity. Bone marrow (**D**) and inflammatory BAL (**E**) neutrophils were isolated from WT (white bars) and myeloid-specific *Phd2^–/–^* (black bars) mice. ECARs were determined by Seahorse (**D**) and glycolytic flux by quantification of ^3^H_2_O release following uptake of 5-^3^H glucose (**E**) in the presence or absence of glucose (1 mg/ml), fMLP (10 μM), and LPS (100 ng/ml) under conditions of normoxia (N, 21% O_2_) and hypoxia (H, 3% O_2_). (**F**) Box plot analysis of the relative change in glycolytic intermediary abundances measured by LC-MS in inflammatory BAL neutrophils from WT and myeloid-specific *Phd2^–/–^* mice following 6 hours culture under conditions of normoxia (N, 21% O_2_) and hypoxia (H, 3% O_2_). Data represent mean with minimum to maximum values, *n* = 3. Overall significance between genotypes for relative analyte abundance was determined by 2-way ANOVA, *P* < 0.5 for WT normoxia (dashed line) vs. *Phd2* normoxia. *P* values obtained via unpaired *t* test (**B** and **E**) and 2-way ANOVA (**D** and **F**).

**Figure 4:**
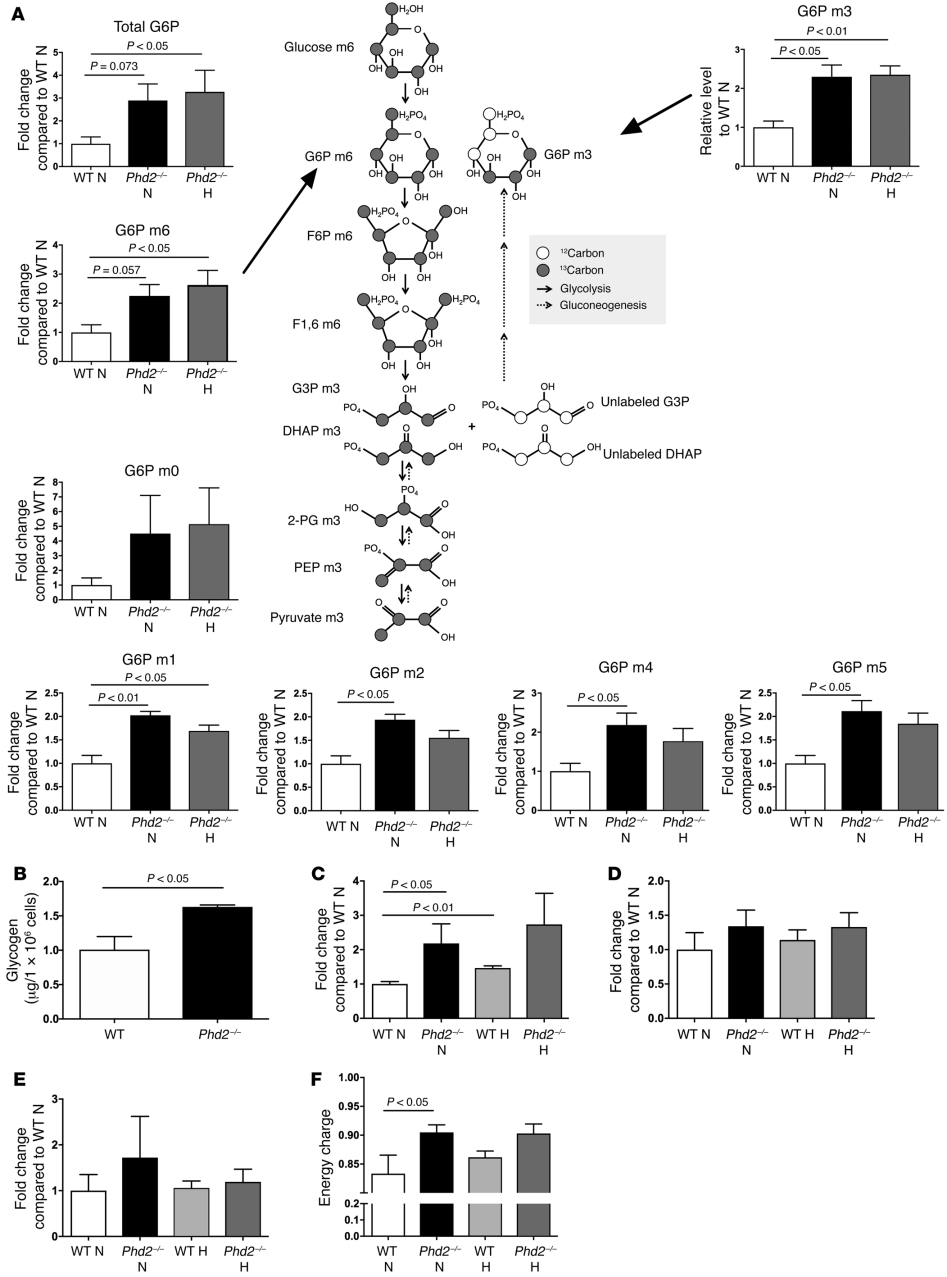
Inflammatory neutrophils deficient in *Phd2* display enhanced glycolytic capacity and glycogen storage with parallel increases in ATP production and utilization. Murine inflammatory BAL neutrophils from WT (white bars) and *Phd2^–/–^* (black bars) mice were studied following isolation 24 hours after in vivo challenge with nebulized LPS (3 mg). (**A**) Glycolytic metabolites. Murine inflammatory BAL neutrophils were cultured in the presence of U-^13^C glucose for 6 hours under conditions of normoxia (21% O_2_) and hypoxia (3% O_2_). Incorporation into G6P and redistribution of ^13^C carbons derived from U-^13^C glucose were measured using LC-MS. Data are presented as relative metabolite abundance and show mean ± SEM, *n* = 3. A diagram of the glycolytic and gluconeogenesis pathways depicting 2 possible outcomes resulting in the generation of G6P m3 isotopomer by recycling of ^13^C carbons is included. Total G6P is a measure of both unlabeled ^12^C G6P and all other mass isotopomers of G6P denoted m0–m6, where m0 contains only ^12^C carbons and m6 with all 6 carbons ^13^C. (**B**) Glycogen content. WT (white bars) and myeloid-specific *Phd2^–/–^* (black bars) inflammatory BAL neutrophils were lysed at time of isolation and following 6 hours culture in normoxia or hypoxia and intracellular glycogen stores quantified by a colorimetric assay. Data represent mean ± SEM, *n* = 5. (**C**–**F**) Energetics. BAL neutrophils were cultured for 6 hours ex vivo and relative ATP (**C**), ADP (**D**), and AMP (**E**) levels determined by LC-MS, enabling calculation of energy charge (**F**). Data represent mean ± SEM, *n* = 3. DHAP, dihydroxyacetone phosphate. *P* values obtained via unpaired *t* test.

To investigate whether *Phd2*-deficient phenotypes could be, at least in part, due to compensatory overexpression of genes encoding for other PHD family members, relative quantification of *Phd* mRNA was undertaken on freshly isolated BAL neutrophils 24 hours after LPS challenge. No differences in expression of either *Phd1* or *Phd3* were observed at an mRNA level (Figure 3A). No changes in either *Hif1a* or *Hif2a* mRNA were observed (Figure 3A), but key regulators of glycolysis and known HIF target genes *Pkm2*, *Pgk*, *Tpi1*, and *Gapdh* were increased (Figure 3B), with a parallel and sustained increase in tissue myeloid cell expression of HIF-1α protein in *Phd2^–/–^* mice (Figure 3C). In light of the observed increase in glycolytic enzymes, we questioned whether *Phd2*-deficient neutrophils would display either a basal increase in glycolysis or an exaggerated metabolic response to proinflammatory mediators. Extracellular acidification rates (ECAR), an indirect measure of glycolysis (28), were initially determined for WT cells, comparing bone marrow isolated neutrophils (Supplemental Figure 4A) with BAL neutrophils (Supplemental Figure 4B). Both immature and inflammatory neutrophil populations displayed detectable baseline ECAR, which could be further increased by supplementation with glucose or stimulation with the chemotactic peptide fMLP (Figure 3D). Baseline glycolytic flux was subsequently determined by measure of ^3^H_2_O release following uptake of 5-^3^H glucose in the presence or absence of LPS (Supplemental Figure 4C). Conditional loss of *Phd2* resulted in a significant increase in bone marrow neutrophil ECAR following stimulation with glucose (Figure 3D) and in BAL neutrophil glycolytic flux (Figure 3E). Analysis of glycolytic intermediaries carried out by liquid chromatography–mass spectrometry (LC-MS) verified the global increase in glycolytic metabolites in the absence of *Phd2* in normoxia, with augmentation of metabolite fold change following exposure of *Phd2*-deficient BAL neutrophils to hypoxia (Figure 3F). In keeping with enhanced availability of glycolytic intermediaries, increased abundance of pentose phosphate pathway metabolites was also seen (Supplemental Figure 4D). Inhibition of the pentose phosphate pathway in untreated and LPS-stimulated WT BAL neutrophils using the NADP^+^-dependent enzyme 6-phosphogluconate dehydrogenase inhibitor 6-aminonicotinamide (6-AN) had no observed effect on neutrophil apoptosis (Supplemental Figure 4E). To investigate whether the increase in glycolytic intermediaries was a consequence of increased glucose uptake or altered metabolic processing of glucose, BAL neutrophils were cultured with uniformly labeled U-^13^C glucose, in which all carbons of a glucose molecule are substituted with a ^13^C isotope (29). This revealed an increase in glucose label redistribution in *Phd2*-deficient BAL neutrophils under conditions of both normoxia and hypoxia. While the increase in total glucose-6-phosphate (G6P) was in keeping with increased glucose uptake (Figure 4A), the presence of multiple G6P isotopomers (m1–m6) (Figure 4A, relative abundance shown in Supplemental Figure 5) demonstrated a measure of conversion of nonglucose substrates into glucose in *Phd2*-deficient BAL neutrophils, an observation further validated by a parallel increase in intracellular glycogen stores (Figure 4B). This functional increase in glucose uptake occurred in the context of unchanged *Glut1* (Supplemental Figure 6A) and *Glut3* (Supplemental Figure 6B) mRNA expression. The global consequences of enhanced glycolytic flux and glycogen stores for neutrophil energetics, ATP production, and utilization were therefore determined. Compared with WT cells, *Phd2*-deficient neutrophils contained higher relative levels of ATP (Figure 4C) and equivalent levels of ADP (Figure 4D) and AMP (Figure 4E), reflecting increased nucleotide production, with an overall increase in cellular energy charge also observed (Figure 4F).

### Inhibition of glycolysis and glucose availability rescues the enhanced neutrophilic responses observed in myeloid-specific Phd2^–/–^ mice.

**Figure 5:**
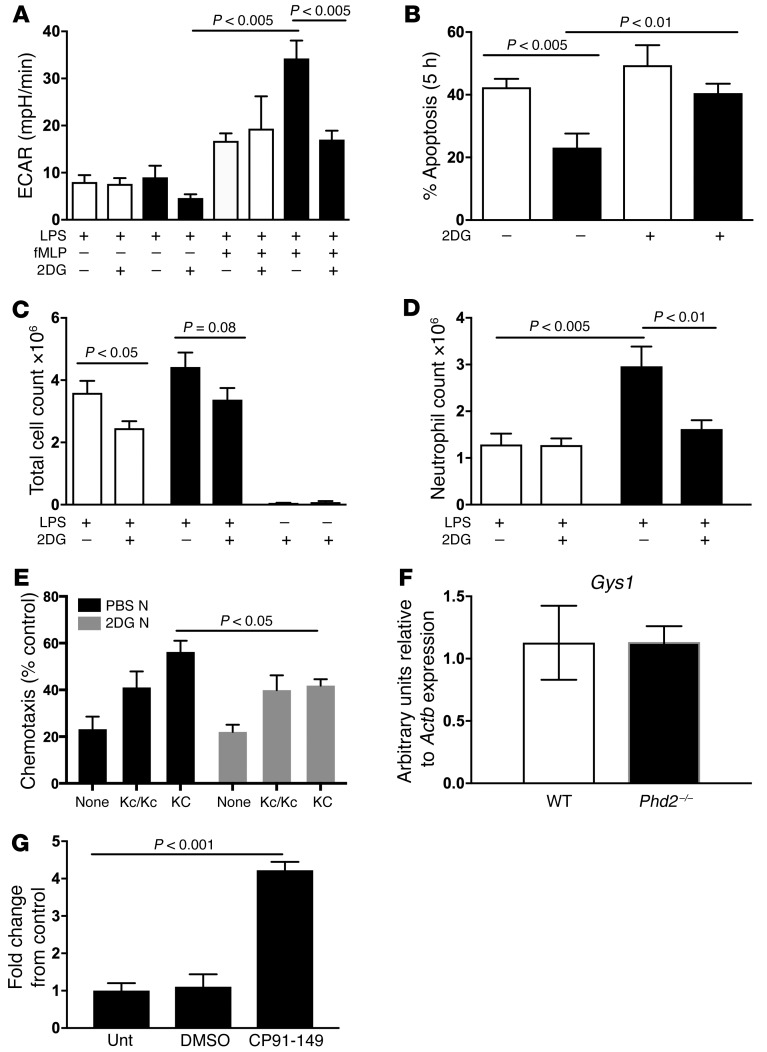
Enhanced neutrophilic inflammation in myeloid-specific *Phd2^–/–^* mice is rescued by inhibition of glycolysis and glucose availability. (**A**) Validation of 2DG. WT (white bars) and myeloid-specific *Phd2^–/–^* (black bars) mice were challenged with nebulized LPS (3 mg) and, 24 hours after challenge, further treated with either i.p. 2DG (500 mg/kg) or PBS control. Forty-eight hours after LPS challenge, mice were sacrificed and bone marrow neutrophils harvested for Seahorse quantification of ECARs, with additional fMLP stimulation ex vivo. (**B**) Apoptosis. Peripheral blood neutrophils were isolated from WT (white bars) and myeloid-specific *Phd2^–/–^* (black bars) mice and apoptosis rates assessed at 5 hours in the presence or absence of 2DG (100 μM). (**C**) In vivo inflammation resolution. WT (white bars) and *Phd2^–/–^* cre^+^ (black bars) mice were challenged with nebulized LPS (3 mg) and 24 hours after challenge further treated with either i.p. 2DG (500 mg/kg) or PBS control. Forty-eight hours after LPS challenge, mice were sacrificed and cells harvested by BAL for total cell counts (**C**) and neutrophil differential counts (**D**). Data represent mean ± SEM, *n* = 5. (**E**) WT mice were challenged with nebulized LPS (T_0_) (3 mg), and i.p. installation with 2DG (500 mg/kg) or PBS vehicle control was carried out at T_10_. Mice were sacrificed at 24 hours (T_24_) and cells harvested by BAL. Chemotaxis of the freshly isolated BAL neutrophils to KC was determined ex vivo using neuroprobe chambers. Data represent mean ± SEM, *n* = 6. (**F**) Glycogen synthase 1 (*Gys1*) expression in bone marrow neutrophils. FACS-sorted bone marrow neutrophils of LPS-nebulized WT and *Phd2-*deficient mice were lysed and TaqMan analysis of cDNA performed with data normalized to β-actin expression. Data represent mean ± SEM, *n* = 4. (**G**) *Phd2-*deficient inflammatory BAL neutrophils were incubated for 20 hours in the presence or absence (Unt) of DMSO vehicle control or the glycogen phosphorylase inhibitor CP-91149. Effects on neutrophil apoptosis were assessed by morphology. Data represent mean ± SEM (*n* = 4). *P* values obtained via unpaired *t* test (**A**–**E**) and 1-way ANOVA (**G**).

**Figure 6:**
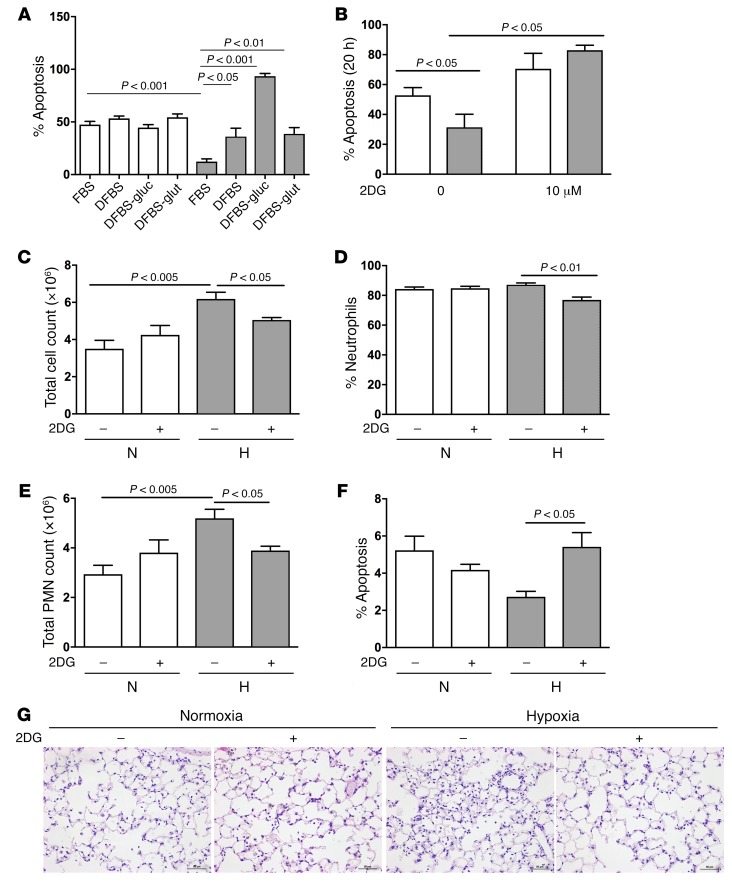
Glycolytic inhibition reverses hypoxic neutrophil survival and persistence in the setting of systemic hypoxia following glycolytic inhibition. (**A** and **B**) Apoptosis. Human peripheral blood neutrophils were cultured ex vivo for 20 hours in the presence or absence of dialyzed FBS and glucose-free media (**A**) or 2DG (**B**) and apoptosis assessed by morphology. DFBS-glu, dialyzed fetal bovine serum-glucose free media. DFBS-glut, dialyzed fetal bovine serum-glutamine free media. (**C**–**F**) In vivo administration of 2DG. WT mice were challenged with nebulized LPS (T_0_) (3 mg). Six hours after installation (T_6_), mice were either maintained in normoxia (21% O_2_) or at 10% O_2_ over an hour prior to i.p. installation at T_10_ with 2DG (500 mg/kg) or PBS vehicle control. Mice were sacrificed at 24 hours (T_24_) and BAL total cell counts (**C**), neutrophil differential counts (**D**), neutrophil total counts (**E**), and neutrophil apoptosis counts (**F**) performed or lungs harvested, fixed with 10% buffered formalin, paraffin-embedded, and sections stained with H&E (**G**). Original magnification, ×400. Data represent mean ± SEM, *n* = 5, with significance determined by one-way ANOVA.

Clinically useful strategies for selectively targeting neutrophil-mediated inflammatory responses are currently lacking. We hypothesized that the uplift in glycolytic capacity seen in *Phd2*-deficient neutrophils was directly responsible, or at least critical for, the exaggerated neutrophil responses seen following challenge with live bacteria or bacterial products and thus represents a therapeutic target. Mice were initially challenged with intratracheal LPS for 24 hours prior to i.p. administration of the glycolytic inhibitor 2 deoxy-d-glucose (2DG). The dosing regimen for 2DG was validated by the observed suppression of bone marrow neutrophil ECAR by in vivo treatment with 2DG (Figure 5A), and the consequences for neutrophil apoptosis and inflammation resolution were then determined. The enhanced survival of peripheral blood neutrophils lacking *Phd2* expression was completely reversed by treatment with 2DG (Figure 5B). Moreover, in vivo administration of 2DG abrogated the persistent increase in BAL total cell counts (Figure 5C) and neutrophil differential counts (Figure 5D). BAL neutrophils isolated from mice treated with 2DG also revealed a reduction in their chemotactic capacity to KC (Figure 5E). To assess whether neutrophils have the capacity to utilize glycogen for survival responses, we first questioned whether they express glycogen synthase, the key enzyme required for glycogen synthesis. Relative quantification of *Gys1* mRNA was undertaken on bone marrow neutrophils and revealed expression of the transcript in both WT and *Phd2^–/–^* cells (Figure 5F). In vitro, inhibition of glycogen breakdown in *Phd2-*deficient inflammatory BAL neutrophils with CP-91149 (a glycogen phosphorylase inhibitor) resulted in significantly increased apoptosis rates (Figure 5G). Together, these data support the concept that changes in the metabolic status of neutrophils critically regulate their function and survival. To investigate the importance of glycolytic capacity for inflammation resolution in the context of hypoxia, human peripheral blood neutrophils were cultured ex vivo and apoptosis determined both in deplete culture conditions and with the glycolytic inhibitor 2DG. Hypoxic survival was abrogated by the absence of glucose (Figure 6A) and by glycolytic inhibition (Figure 6B). In vivo treatment with 2DG during the resolution phase of the inflammatory response resulted in rescue of the persistent inflammation seen in the presence of hypoxia (Figure 6, C–E, G) with return of neutrophil apoptosis rates to normoxic levels (Figure 6F).

### Pan hydroxylase inhibition replicates the Phd2-deficient phenotype in vivo.

**Figure 7:**
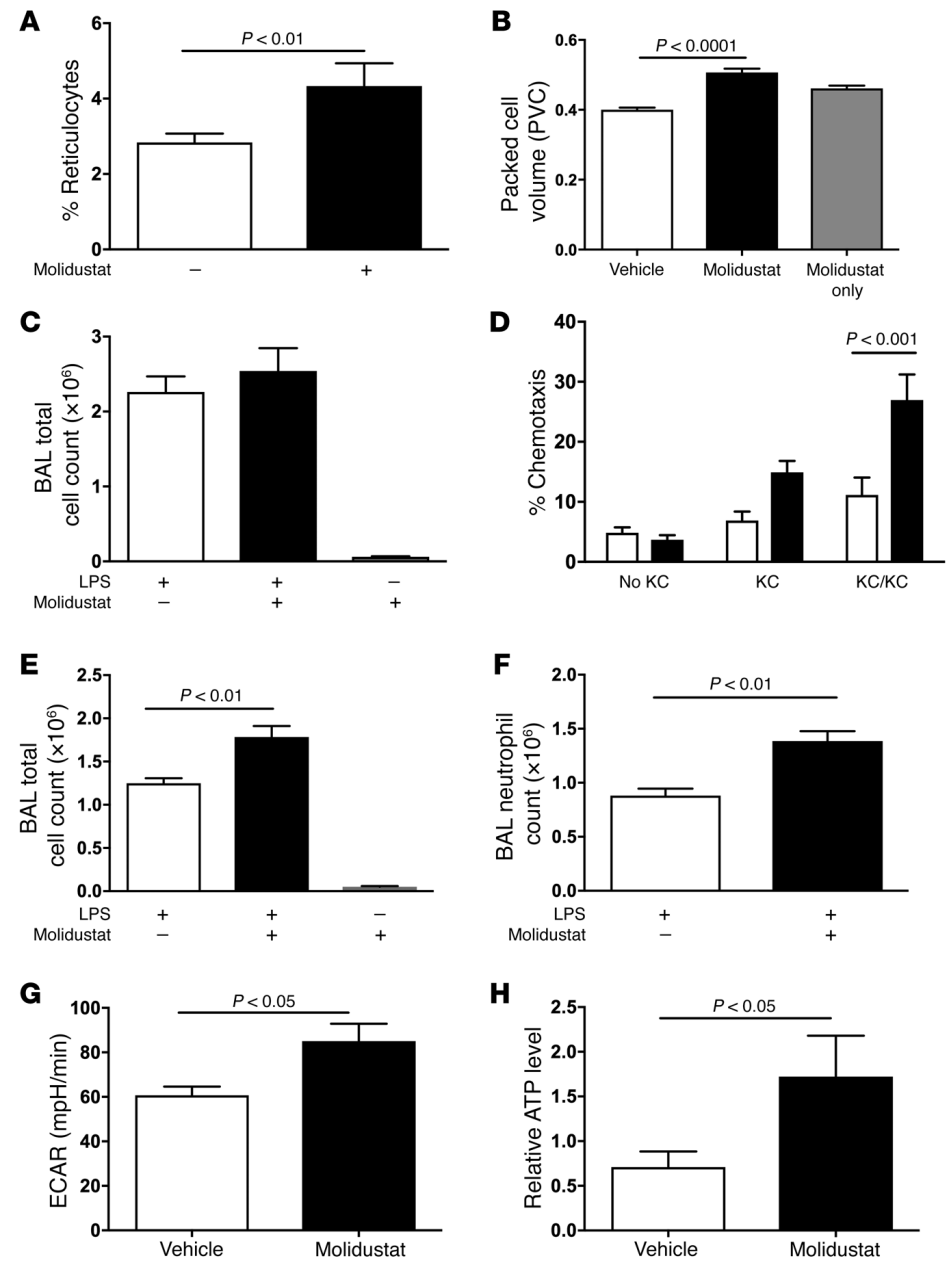
Non-PHD isoform-selective inhibition in vivo replicates the *Phd2*-deficient phenotype of augmented neutrophilic inflammation. WT mice were treated with the HIF-prolyl hydroxylase inhibitor molidustat (5 mg/kg) or vehicle control (DMSO) by gavage 2 hours (T_–2_) prior to challenge with nebulized LPS (3 mg) (T_0_). (**A** and **B**) Whole blood was harvested at T_48_ for percentage of reticulocyte counts (**A**) and packed cell volume measurements (**B**). (**C** and **D**) Recruitment. Mice were sacrificed 6 hours after LPS installation (T_6_) and total cell counts in BAL were determined (**C**). The chemotactic mobility of neutrophils recovered from the BAL toward KC was determined ex vivo using neuroprobe chambers (**D**). (**E** and **F**) Resolution. Twenty-four hours after the initial molidustat dosing (T_22_), a second dose of molidustat (5 mg/kg) was administered by gavage and animals sacrificed after a further 22-hour period (T_48_). BAL total cell counts (**E**) and neutrophil (**F**) total counts were performed. (**G** and **H**) Twenty-four hours after LPS challenge, mice (T_24_) were sacrificed and bone marrow neutrophils harvested for Seahorse quantification of ECARs (basal ECAR levels shown) (**G**). ATP levels were measured in BAL neutrophils by LC-MS (**H**). Data represent mean ± SEM of at least 4 independent experiments, with significance determined by unpaired *t* test.

In order to investigate the consequences of in vivo administration of the potent HIF-prolyl hydroxylase inhibitor molidustat (30, 31) for inflammation resolution, molidustat was administered in vivo by gavage 2 hours (T_–2_) prior to challenge with nebulized LPS (3 mg) (T_0_). Molidustat is an example of a family of triacyclic triazole–based PHD inhibitors, which compete with 2-oxoglutarate for binding with the ferrous iron at the active site of the PHDs (32). Molidustat increased both circulating reticulocyte (Figure 7A) and packed cell volume (Figure 7B), confirming expected actions at the concentration used, but did not significantly modify the number of neutrophils recruited to the lung following LPS challenge (Figure 7C) despite an increase in basal motility (Figure 7D). However, molidustat significantly delayed inflammation resolution, with an increase in total BAL cells (Figure 7E) reflecting an increase in the number of neutrophils persisting at 48 hours (Figure 7F). In addition, in vivo administration of molidustat resulted in an increase in baseline bone marrow neutrophil ECAR (Figure 7G) and significantly increased ATP levels in BAL neutrophils (Figure 7H).

## Discussion

A tightly coordinated innate immune response is critical for effective pathogen clearance with limited collateral tissue damage. There is a clinical need for therapies that selectively target excessive or inappropriate inflammation while maintaining effective antimicrobial host responses. In this work, we identify an important role for PHD2 in the regulation of neutrophilic inflammation. We observe that myeloid-specific loss of *Phd2* results in a disordered inflammatory response that is detrimental to the host and imparts no measured benefit for bacterial killing or clearance. In marked contrast to the HIF-independent phenotype of abrogated hypoxic survival in *Phd3*-deficient neutrophils (11), loss of *Phd2* results in a delay in both neutrophil apoptosis and inflammation resolution. In parallel with enhanced neutrophil survival, we also observed an intrinsic neutrophil phenotype of augmented recruitment, enhanced chemotaxis, and an increase in the functional reserve capacity of aged neutrophils. Given neutrophil migration is a highly energy-requiring process (33), that neutrophils are unusual in their capacity to predominantly utilize glycolysis for ATP production (34), and that glycolysis enables much more rapid access to ATP than other metabolic processes (35), the glycolysis-driven augmented function is energetically favorable. The mechanisms that drive this glycolytic response and its functional consequences do, however, require further discussion. The increase in energy charge in *Phd2*-deficient neutrophils suggests that an increased demand for and utilization of ATP, as reflected in the enhanced chemotactic capacity of these cells, is fully matched by the augmented flux through glycolysis. Interestingly, the increase in glycolytic flux occurs in the setting of increased G6P production. We observed an increase in both m6 fully labeled and m5–m0 partially labeled and unlabeled G6P fractions, respectively, following culture with U^13^-C. This identifies first that *Phd2*-deficient neutrophils are capable of increased glucose uptake and utilization where glucose is freely available, and second, that neutrophils are capable of the conversion of nonglucose substrates into glucose. This has particular relevance to the in vivo state, and inflamed sites in particular, given the limited glucose availability. While previous electron microscopy (EM) studies have detailed the presence of glycogen bodies within quiescent neutrophils (36, 37), our work provides direct evidence for regulation of neutrophil apoptosis through glycogen availability. The wider implications of the ability of neutrophils to maintain intracellular glucose and glycogen stores also require consideration, given the previous descriptions of neutrophil dysfunction in association with G6PT deficiency in humans (38) and G6Pase-b inactivation in mice (39). Furthermore, this leads us to speculate that neutrophils can themselves undergo gluconeogenesis and that PHD2 is an important regulator of this process. It is of particular interest, given the recent observation that both liver-specific knockdown of *Phd2* and oral administration of a nonisoform-selective PHD inhibitor GSK360A enhanced lactate-glucose recycling between the muscle and the liver and improved survival in an endotoxic shock model (40). Since the myeloid cell response to an endotoxin challenge predominates in the acute response, this raises important questions as to the potential for neutrophils to undergo gluconeogenesis and the role of gluconeogenesis in the systemic response to infection.

To address whether the exaggerated inflammatory response observed in *Phd2*-deficient mice was modulated by the observed increase in glycolytic capacity and amenable to therapeutic manipulation in vivo, animals were treated with i.p. 2DG 24 hours after challenge with nebulized LPS. Despite the potential global consequences for the host of glycolytic inhibition, in vivo blockade of glycolysis resulted in abrogation of exaggerated neutrophil inflammatory responses and survival in the myeloid-specific *Phd2*-deficient mice. This led us to question whether there were other situations in which neutrophils demonstrate suppressed PHD activity and in which the innate immune response could be modified by inhibiting glycolysis. We used the combination of nebulized LPS and systemic hypoxia, given we have previously described the activation of HIF-1α in neutrophils in this setting (11) and would predict this to occur in the setting of suppressed PHD2 activity. Administration of the glycolytic inhibitor 2DG during the resolution phase of the lung injury response completely abrogated both hypoxic neutrophil survival and the sustained inflammatory response. This observation suggests that the therapeutic targeting of neutrophil glycolysis may represent a novel approach to limiting inappropriate or persistent neutrophilic inflammation in situations where increased HIF-1α activity is observed.

Finally, the broader consequences of long-term non-PHD isoform-selective inhibition for outcomes of the innate immune response require consideration. Our in vivo data would suggest that pan hydroxylase inhibition with molidustat results not only in enhanced neutrophil functional responses with increased basal motility and chemotaxis, but also in delayed inflammation resolution. This raises important questions as to the consequence of unchecked HIF activity in the setting of PHD inactivation and represents an important divergence from the normal physiological state in which PHD2 expression follows HIF transcriptional activation. Our observation, while providing important supportive evidence for the dominance of *Phd2* in neutrophils in normoxia, also highlights the potential for disordered neutrophil-mediated inflammatory responses following treatment with non-PHD isoform selective inhibitors and suggests a degree of caution when considering the long-term clinical use of pan hydroxylase inhibitors (30) in the treatment of chronic anemia. Our data are also further demonstration of the need for the development of more selective targeting of individual PHD enzymes in the clinical arena.

## Methods

### Animals

Lysozyme M–driven Cre (*LysM-Cre*) targeted *Phd2* deletions to myeloid lineage cells with animals backcrossed to a C57BL/6 background (10). *Phd2^fl/fl^ LysM-Cre^–/–^* littermates were used as WT controls. Mice with *MRP8*-driven Cre-targeted *Phd2* deletion (*Phd2^fl/fl^ MRP8-Cre^+/–^*) were generated by crossing *Phd2^fl/fl^* animals with the previously reported neutrophil-specific Cre driver *MRP8* (27). For DSS colitis, C57BL/6 WT and previously described whole animal heterozygous *Phd2^+/–^* animals were studied (41). Mice with conditional tamoxifen-induced deletion of *Phd2* (*Phd2^Rosa26CreERT2;fl/fl^*) were used in the study of neutrophil energy states and glycolytic capacity (10). Alveolar macrophage depletion was performed using 48 hours pretreatment with clodronate, as previously described (25).

### Intratracheal pneumonia model

WT C57BL/6 mice were anesthetized with ketamine (100 mg/kg i.p.; Vetalar V, Pfizer) and acepromazine (5 mg/kg i.p.; Calmivet Solution Injectable, Vetoquinol). The fur was shaved from the neck and a small incision made. The trachea was then exposed by blunt dissection and cannulated with a 24-gauge cannula (Jelco Radiopaque Cannula, Smiths Medical International Ltd.). Each mouse then had 1 × 10^7^ CFU of TIGR4 instilled via the trachea. At 14 hours, BAL was performed via cannulation of the trachea. Total cell counts were calculated using hemocytometer counts and differential counts assessed on cytocentrifugation slides. Levels of IgM were analyzed using commercially available kits (Mouse IgM ELISA Quantitation Set, Bethyl Laboratories Inc.).

### Quantification of viable bacterial counts

Ten-fold serial dilutions were performed on whole blood aliquots. Three 10 μl drops from each of 6 dilutions were then plated onto blood agar plates and cultured overnight in 37°C to calculate viable bacterial counts. After collection of the BAL fluid, the lungs were carefully dissected and stored in sterile tubes. The lungs were homogenized and 10-fold serial dilutions performed on each sample to calculate viable bacterial counts, which were normalized to count per pair of lungs.

### LPS acute lung injury model

Direct tracheal instillation of bacterial LPS (0.3 mg) was performed on anesthetized mice. For animals receiving treatment with 2DG, LPS (3 mg) was administered via nebulization, enabling subsequent i.p. administration of either 2DG (500 mg/kg) or PBS vehicle control (42). Mice were sacrificed at 6, 24, and 48 hours (43), tracheas recannulated, and lungs instilled with 3.5 ml of ice-cold PBS in 0.5 ml aliquots. Hemocytometer counts were performed on the recovered BAL samples, which were then pelleted (1000 *g*, 5 minutes, 4°C) and resuspended in FCS prior to cytocentrifugation for differential cell counts and morphologic scoring of apoptosis. Chemokine/cytokine concentrations in lavage samples were determined by BD cytometric bead array using BD FACSArray acquisition software. Limits of detection were 10–2500 pg/ml.

### DSS-induced acute colitis model

Colitis was induced using a previously described method (44) and mice sacrificed at day 6. Sections of intestine were stained with anti-MPO antibody following deparaffinization, and total neutrophils per high-power field (hpf) averaged from 5 fields per section.

### Neutrophil isolation and culture

Murine inflammatory BAL neutrophils were isolated 24 hours after challenge with LPS (0.3 mg), with peripheral blood neutrophils isolated using negative magnetic selection and bone marrow neutrophils isolated by discontinuous Percoll gradients. Cells were cultured for 5–20 hours in normoxia (19 kPa) or hypoxia (3 kPa) at 5% CO_2_, as previously described (11).

### Neutrophil functional assays

#### Chemotaxis.

5 × 10^4^ neutrophils were plated on a semipermeable membrane (ChemoTx Chemotaxis System, pore size 5 μm, Neuroprobe) in the presence or absence of KC at T_0_ and following 6 hours of aging, incubated for 1 hour at 37°C, spun at 300 *g* for 10 minutes, and hemocytometer counts performed. The number of cells in each well was expressed as a percentage of the total number of cells loaded into each well.

#### Respiratory burst.

Neutrophils (1 × 10^6^/ml) were cultured with 5 μM 2′,7′-dichlorofluorescin diacetate (DCF) for 30 minutes, stimulated for 30 minutes with 10 μM fMLP (Sigma-Aldrich), and FL1 geometric mean fluorescence determined by flow cytometry.

#### Inner mitochondrial membrane potential.

Neutrophils (1 × 10^6^/ml) were resuspended in 3 nM TMRM (Life Technologies) dissolved in 1× HBSS, with and without 10 μM carbonyl cyanide 3-chlorophenylhydrazone (CCCP) (Sigma-Aldrich), and FL2 geometric mean fluorescence determined by flow cytometry.

#### Mitochondrial ROS production.

Neutrophils (1 × 10^6^/ml) were resuspended in 5 μM MitoSOX (Life Technologies) dissolved in 1× DPBS, with and without 10 μM CCCP (Sigma-Aldrich), and FL2 geometric mean fluorescence determined by flow cytometry.

#### Phagocytosis.

1 × 10^6^ inflammatory BAL neutrophils were incubated with Alexa Fluor 488–conjugated *E*. *coli* BioParticles (Thermo Fisher Scientific) (MOI 10:1) for 1 hour. Ice controls were included to account for bacterial adherence as opposed to internalization. Cells were washed with ice-cold PBS, resuspended in FACS buffer, and analyzed by flow cytometry. Data analysis was carried out with FlowJo version 10.

### Flow cytometry

Mouse BAL cells were treated with α-CD16/32 Fc block (eBioscience) and mouse serum (Thermo Fisher Scientific) prior to staining with antibodies. Relevant full minus one (FMO) samples for each group were used as controls. Antibodies used were as follows: Ly6G (1A8, BioLegend), CD11a (M17/4, BioLegend), CD11b (M1/70, BioLegend), CXCR2 (SA044G4, BioLegend), and CXCR1 (FAB8628A-025, RnD). Live cells were gated following staining with DAPI (Invitrogen) prior to acquisition. BAL neutrophils were gated according to Ly6G^+^ and forward scatter (FSC)/side scatter (SSC) properties. Cells were acquired on an LSRFortessa (BD). Compensation was performed using BD FACSDiva software and data analyzed with FlowJo version 10.

### Isolation, culture, and functional assays of BMDM

Red blood cell lysis was carried out on whole bone marrow cells from naive WT and *Phd2*-deficient mice. Cells were cultured in Glutamax DMEM supplemented with 1% penicillin/streptomycin, 10% FBS, and 20% L929 medium. Successful differentiation following 7 days of culture was determined by FACS staining for the macrophage marker F4/80.

#### Phagocytosis.

Alexa Fluor 488–conjugated *E*. *coli* BioParticles (MOI 1:1) were administered to cells for 1 hour. Following vigorous washing with PBS, phagocytosis of *E*. *coli* was measured using flow cytometry. Data analysis was carried out with FlowJo version 10.

#### Efferocytosis.

Differentiated BMDM cells were incubated with PKH26-stained apoptotic human neutrophils (cultured for 20 hours in normoxic conditions) at a ratio of 5:1 for 1 hour. Following vigorous washing with PBS, uptake of apoptotic human neutrophils was assessed by flow cytometry. Data analysis was carried out with FlowJo version 10.

### RNA isolation and relative quantification

Murine BAL leukocytes (1 × 10^6^/condition) were lysed and RNA extracted using the mirVana Total RNA Isolation Protocol (Ambion). Samples were treated with DNase (Ambion) and random hexamer cDNA synthesized by reverse transcription. Assays-on-Demand Gene Expression TaqMan MGB 6FAM dye–labeled products (Applied Biosystems) were used for relative quantification of cDNA.

### Validation of *Phd2* transcript knockdown

For *Phd2^fl/fl^ LysM-Cre^+/–^* mice, Percoll-purified bone marrow neutrophils from *Phd2^–/–^* and WT littermates were FACS sorted based on FSC/SSC. For *Phd2^fl/fl^ MRP8*-Cre^+/–^ mice, following BAL neutrophil isolation from mice challenged with nebulized LPS (3 mg) sacrificed at 48 hours after challenge, cells were subjected to Percoll density centrifugation. Purified cells were lysed, and RNA was extracted and reverse transcribed. Assays-on-Demand Gene Expression TaqMan MGB 6FAM dye–labeled products (Applied Biosystems) were used for relative quantification of cDNA (*Phd2* probe: ACGAAAGCCATGGTTGCTTGTTACCCA; forward: GCTGGGCAACTACAGGATAAAC; reverse: CATAGCCTGTTCGTTGCCT).

### Immunohistochemistry

For histological sections, unlavaged lungs were fixed via the trachea with 10% buffered formalin at 20 cm H_2_O. Paraffin-embedded blocks were prepared and sections stained with anti–HIF-1α (polyclonal; Novus Biologicals), anti–HIF-2α (clone ep 190b; Novus Biologicals), or isotype control following deparaffinization.

### Seahorse

Neutrophils were resuspended in XF assay media at a concentration of 3 × 10^6^/ml. Three million cells per condition were plated onto a XF24 cell plate precoated with Cell-Tak (Corning). Cells were stimulated with fMLP (10 μM) or fMLP plus glucose (1 mg/ml). The oxygen consumption rate (OCR) and ECAR were measured at intervals of 7 minutes over a 90-minute cycle using a Seahorse XF24 (Seahorse Bioscience USA).

### Glycolytic flux

Murine BAL leukocytes (0.5 × 10^6^) were incubated for 6 hours in RPMI 1640 medium (supplemented with 5.5 mM unlabeled glucose, 10% FCS, and 1% penicillin/streptomycin) containing 0.4 μCi/ml [5-^3^H]-d-glucose (PerkinElmer). Cells were pelleted (420 *g* for 10 minutes) and supernatant transferred into glass vials containing 12% perchloric acid sealed with rubber stoppers. ^3^H_2_O was captured in hanging wells containing a piece of Whatman paper soaked with H_2_O over a period of 48 hours at 37°C to reach saturation. Radioactivity in the paper was determined by liquid scintillation counting.

### Intracellular glycogen stores

Murine BAL leukocytes (1 × 10^6^/condition) were lysed with 200 μl ice-cold H_2_O and boiled for 10 minutes at 95 degrees; lysates were centrifuged at 18,000 *g* at 4°C for 10 minutes to remove cell debris and snap frozen. Glycogen concentration was measured by colorimetric assay (BioVision).

### Energy status

A total of 1 × 10^6^ murine BAL leukocytes were harvested in 100 μl of ice-cold 5% PCA supplemented with 1 mM EDTA. ATP, ADP, and AMP levels were measured using ion-pair RP-HPLC. The energy charge was expressed as ([ATP] + 1/2 [ADP]/[ATP] + [ADP] +[AMP]), and the energy status of the cells as the ratio of ATP to ADP content.

### LC-MS

Murine BAL leukocytes (2 × 10^6^/condition) were harvested in 100 μl of 80% methanol. Measurements of relative levels of analyte abundance and 13C incorporation into glycolytic intermediates were performed using a Dionex UltiMate 3000 LC System (Thermo Scientific) coupled to a Q Exactive Orbitrap Mass Spectrometer (Thermo Scientific) operated in negative mode. Practically, 25 μl of sample was injected on a SeQuant ZIC/pHILIC Polymeric column (Merck Millipore). The gradient started with 10% solvent B (10 mM NH4-acetate in mqH_2_O, pH 9.3) and 90% solvent A (acetonitrile) and remained at 10% B until 2 minutes after injection. Next, a linear gradient to 80% B was carried out until 29 minutes. At 38 minutes, the gradient returned to 40% B, followed by a decrease to 10% B at 42 minutes. The chromatography was stopped at 58 minutes. The flow was kept constant at 100 μl/min, and the column was kept at 25°C throughout the analysis. The MS operated in full scan–SIM mode using a spray voltage of 3.2 kV, capillary temperature of 320°C, sheath gas at 10.0, auxiliary gas at 5.0. AGC target was set at 1e6 using a resolution of 140,000, with a maximum IT of 500 ms. Data collection and analysis were performed using Xcalibur Software (Thermo Scientific). Isotope correction was carried out as previously described (45) using an in-house software tool.

### Reducing equivalents

Ratios of oxidized to reduced NADP/NADPH were calculated in murine BAL leukocyte lysates (1 × 10^6^/condition) following quantification by fluorimetric enzyme cycling assay (AbCam).

### Statistics

Significance was determined by unpaired 2-tailed *t* tests unless otherwise stated. Data are expressed as mean ± SEM and are representative of at least 3 independent experiments. *P* < 0.05 was considered significant.

### Study approval

Animal experiments were conducted in accordance with the UK Home Office Animals (Scientific Procedures) Act of 1986. All animal studies were approved by The University of Edinburgh Animal Welfare and Ethical Review Board.

## Author contributions

PS, JAW, HMM, DHD, PC, MKBW, and SRW designed the experiments. PS, JAW, RSD, FM, AJH, AL, AART, HMM, and BG performed the experiments. DHD, CTT, MS, CP, PJR, CJS, PHM, ERC, MM, BG, PC, MKBW, and SRW provided technical expertise and performed data analysis. All authors contributed to writing the manuscript.

## Supplementary Material

Supplemental data

## Version 1 08/14/2017

Electronic publication

## Version 2 09/01/2017

Print issue publication
