# Supplementary material for: Prolyl hydroxylase 2 inactivation enhances glycogen storage and promotes excessive neutrophilic responses
Source: J Clin Invest. 2017 Aug 14;127(9):3407–20. doi: 10.1172/JCI90848 (PMC5669581; doi:10.1172/JCI90848)
Supplement: Supplemental data [file jci-127-90848-s001.pdf]

Supplemental data

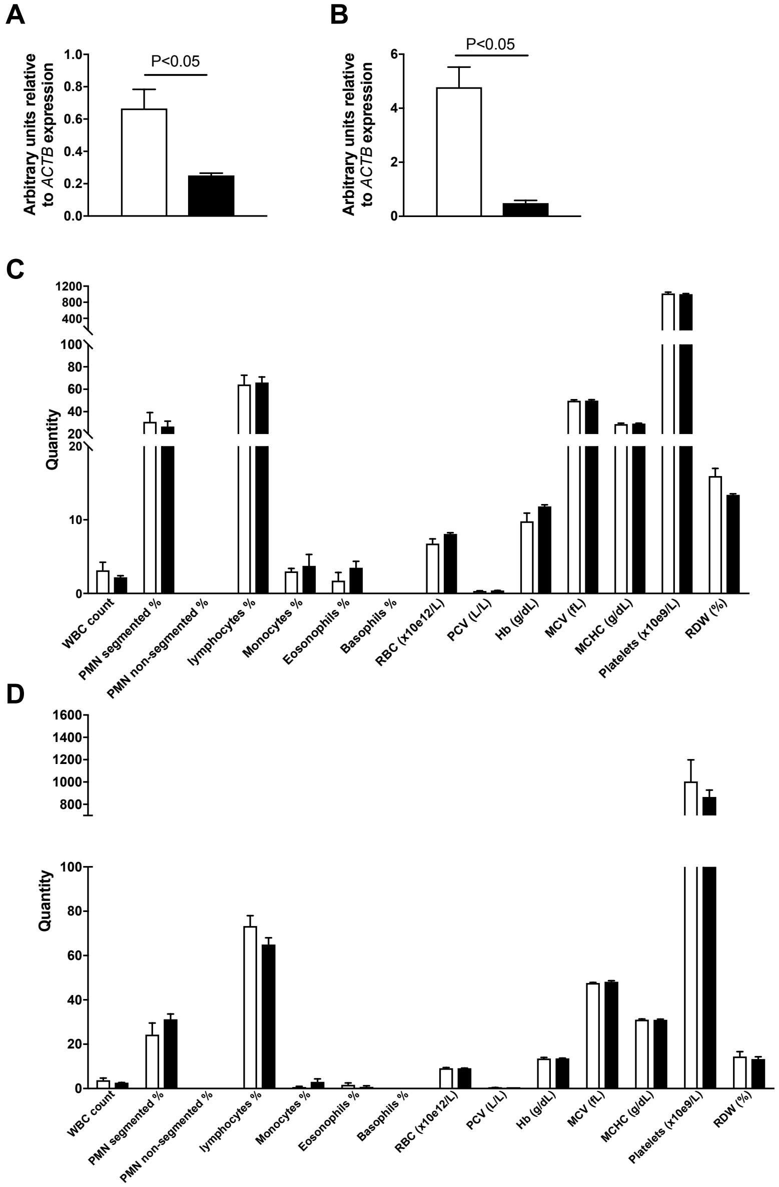

Supplementary figure 1. Myeloid specific *Phd2* deletion does not affect naive and LPS-stimulated total leukocyte and differential cell counts

Phd2 expression in wild-type (wt) (open bars) and myeloid specific *Phd2*$^{-/-}$ (closed bars) bone marrow neutrophils (A) and BMDMs (B). Total RNA was extracted from cells followed by TaqMan analysis of cDNA with data normalised to β-*Actin* expression. Data represents mean ±SEM, n=4 with significance determined by unpaired t test with Welch's correction. (C,D) Baseline and LPS-stimulated differential blood counts. Whole blood samples from naive untreated (C) and LPS nebulised (D) mice were collected in 0.5 M EDTA and subjected to clinical pathology evaluation for a complete blood count. Data represents mean ±SEM (C n=4, D n=3).

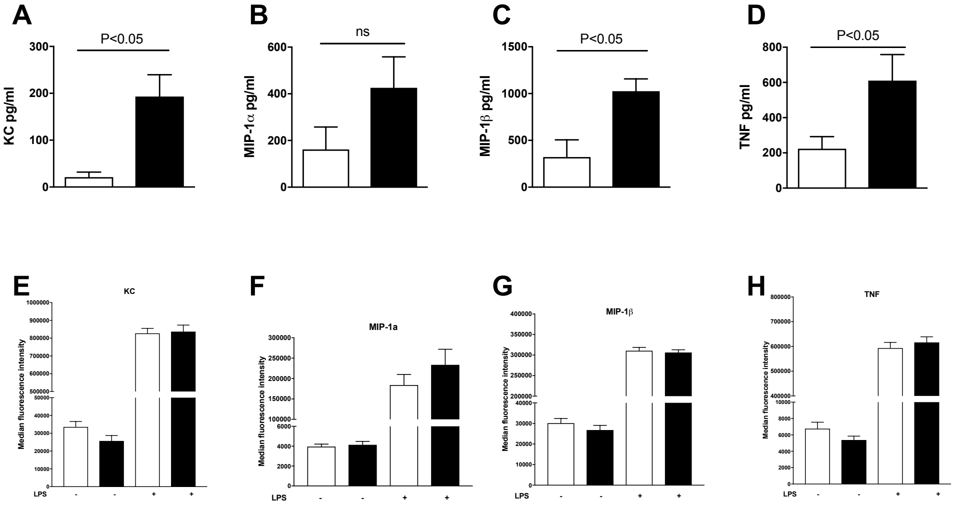

Supplementary figure 2. Myeloid specific deletion of *Phd2* results in a BMDM independent augmented cytokine response to LPS mediated acute lung injury

Wild-type (wt) (open bars) and myeloid specific *Phd2*$^{-/-}$ (closed bars) mice were studied in parallel. (A-D) Acute lung injury. Intra-tracheal LPS (0.3 mg) was instilled in anaesthetised mice, mice sacrificed at 6 hours post challenge and supernatants harvested by bronchoalveolar lavage for measures of KC (A), MIP-1a (B), MIP-1B (C) and TNF (D) release. Data represents mean ±SEM (n=7). Cytokine expression analysis of resting and LPS stimulated wildtype and *Phd2*$^{-/-}$ BMDM cells (E-F). BMDM cells were cultured for 24 h ± LPS. Supernatants were harvested for measure of KC (E), MIP-1a (F), MIP-1B (G) and TNF (H) release. Data represents mean ±SEM (n=6).

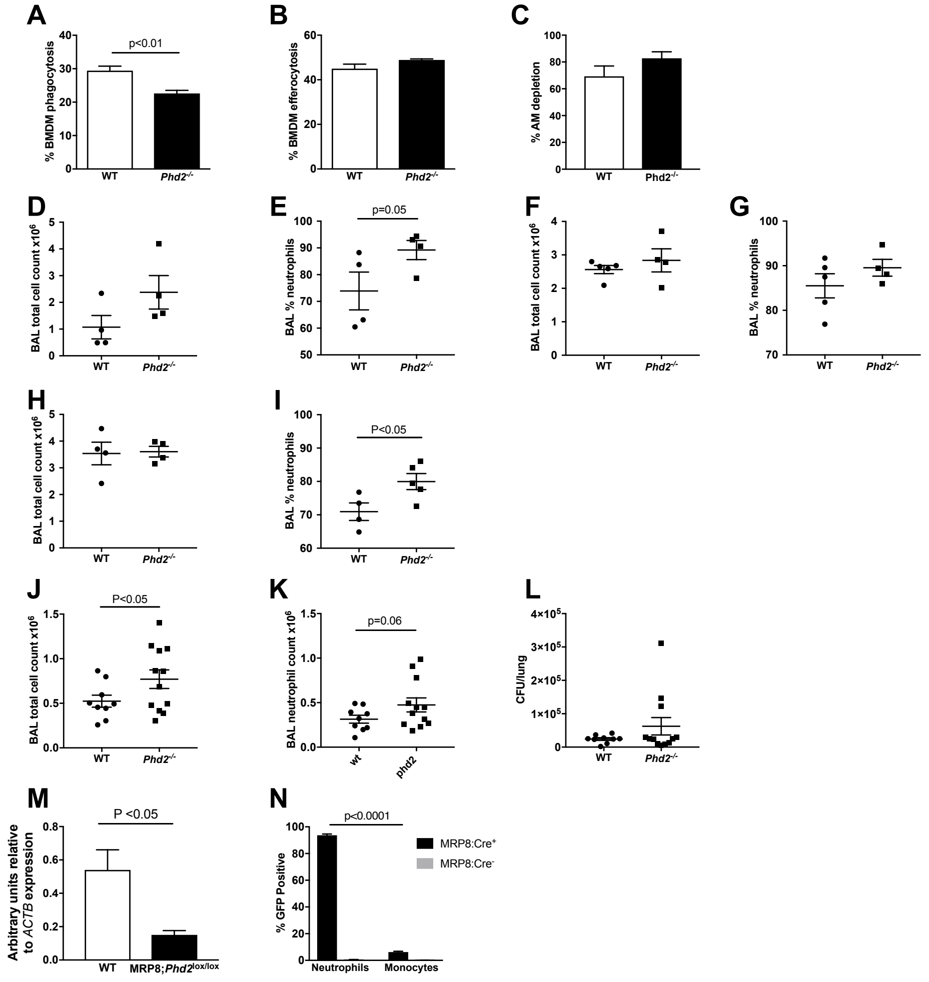

Supplementary figure 3. Increased neutrophilic inflammation with Phd2-deficiency is consequent upon an intrinsic neutrophil phenotype

Analysis of wild-type and *Phd2*$^{-/-}$ BMDM capacity to phagacytose and efferocytose (A,B). BMDMs were incubated with fluorescently labeled *E. coli* (A) or apoptotic human neutrophils (B) for 1 hour. Phagocytosis and efferocytosis rates were measured by flow cytometry. Data represents mean ±SEM (n=4). Assessment of neutrophil responses in alveolar macrophage (AM) depleted mice (C-L). Alveolar macrophage depletion was carried out by intranasal administration of 50 μl of clodronate liposomes and depletion efficiency assessed by flow cytometry (C). Macrophage depleted WT and myeloid specific *Phd2*$^{-/-}$ mice were studied in parallel. Acute lung injury. Mice were nebulised with LPS (3 mg) and sacrificed at 6 hours (D, E), 24 hours (F,G) and 48 hours (H, I) post challenge. Data represents mean ±SEM (n≥4). *Streptococcus pneumoniae* infection. Mice were infected via the trachea with 5x10^{5} cfu of serotype 2 *S. pneumoniae* (D39). Cells were harvested by bronchoalveolar lavage at 20 hours and total cell counts (J) and neutrophil differential counts (K) obtained. Viable bacterial counts were recovered from homogenised lung (L). Data represents mean ±SEM (n≥9). Knockdown of *Phd2* in wild-type and MRP8;*Phd2*$^{lox/lox}$ BAL neutrophils (M,N). Mice were challenged with nebulised LPS (3 mg) and sacrificed at 48 h post challenge. BAL neutrophils were lysed and TaqMan analysis of cDNA performed with data normalised to β-*Actin* expression (M). MRP driven green fluorescent protein (GFP) expression in circulating blood neutrophils and monocytes was used to determine deletion specificity as assessed by flow cytometry (N).

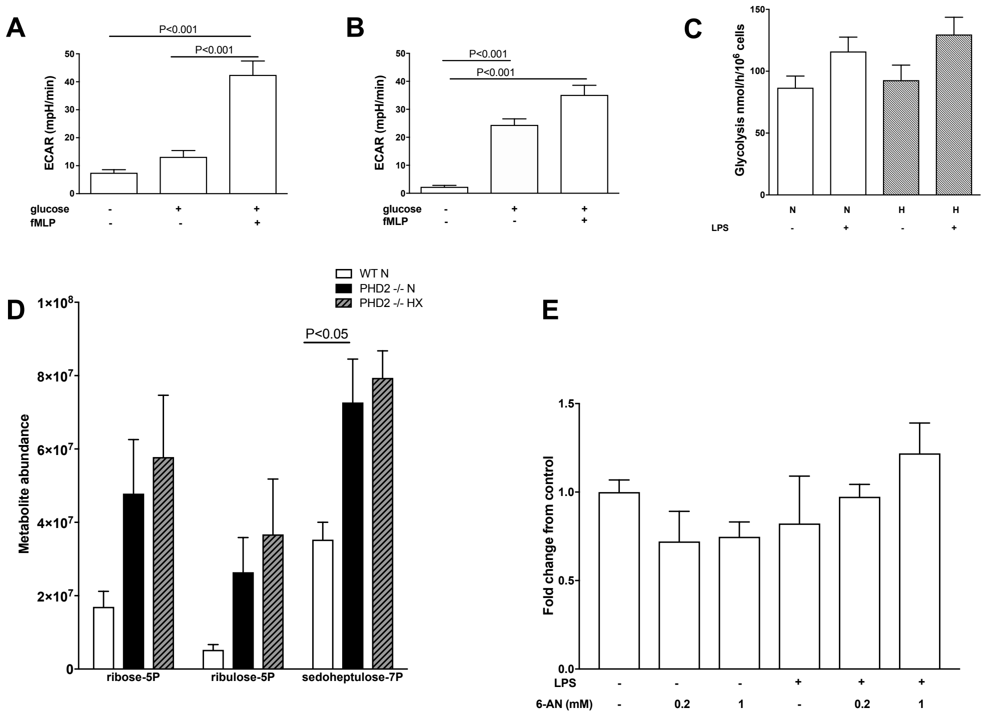

Supplementary figure 4. Metabolic phenotyping of bone marrow and inflammatory BAL neutrophils

(A,B) Seahorse quantification of extracellular acidification rates of wild-type unstimulated murine bone marrow neutrophils (A) and inflammatory BAL neutrophils harvested 24 hours post *in vivo* challenge with nebulised LPS (3mg) (B) was undertaken in the presence or absence of glucose (1mg/ml) and fMLP (10 μM) (n=13 bone marrow, n=17 BAL). (C) Glycolytic flux of wild-type BAL neutrophils. Glycolytic flux was quantified by ^3H_2O release following uptake of 5-^{3}H glucose under conditions of normoxia (N, 21% O_2) and hypoxia (H, 3% O_2) in the presence or absence of LPS (100 ng/ml) (n=3). Data represents mean ±SEM. (D) BAL neutrophils were harvested from wild-type and $Phd2^{-/-}$ mice 24 hours post *in vivo* challenge with nebulised LPS (3mg). Cells were cultured for 6 hours under conditions of normoxia (N, 21% O_2) and hypoxia (H, 0.5% O_2) and total abundance of ribose-5-phasphate (ribose-5p), ribulose-5-phosphate (ribulose-5P) and sedoheptulose-7-phosphate (sedoheptulose-7P) was measured using LC-MS. (E) Inhibition of the pentose phosphate pathway in wild-type inflammatory BAL neutrophils. Cells were incubated for 20 h in the presence or absence of LPS and the pentose phosphate pathway inhibitor 6-AN. Effects on neutrophil apoptosis were assessed by morphology. Data represents mean ±SEM (n=3).

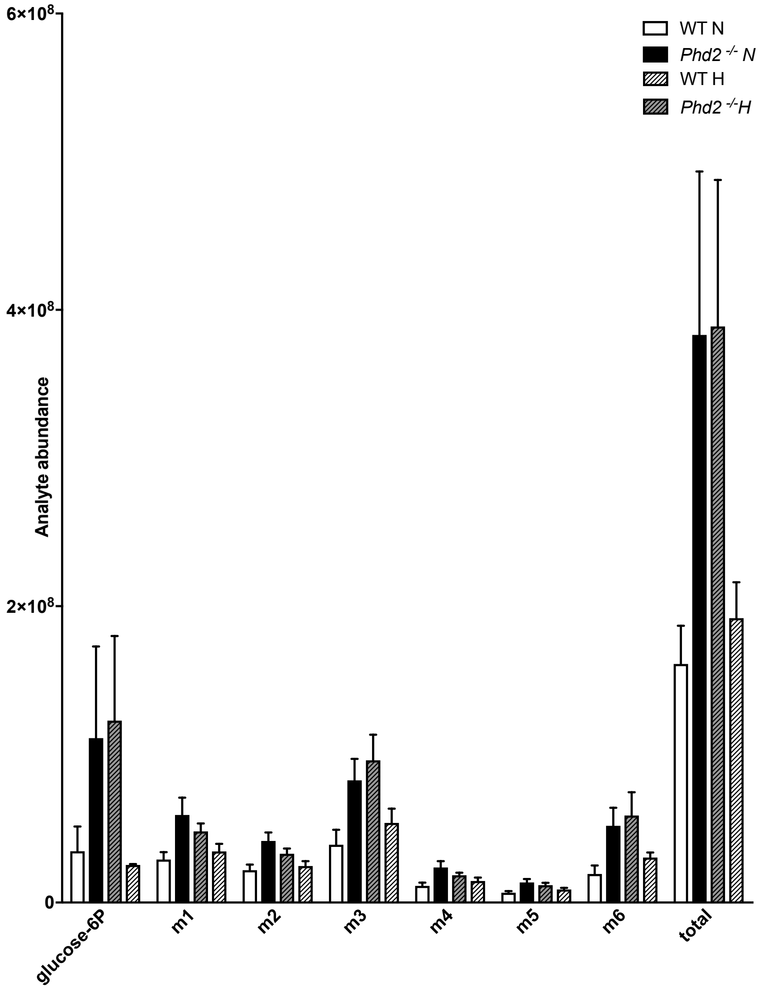

Supplementary figure 5. *Phd2*-deficient inflammatory BAL neutrophils display an increase in relative glucose-6P mass isotopomer abundance comparable to levels found in wild-type cells cultured in hypoxia

BAL neutrophils were harvested from wild-type and *Phd2*$^{-/-}$ mice 24 hours post *in vivo* challenge with nebulised LPS (3mg). Cells were cultured in the presence of U-^{13}C glucose for 6 hours under conditions of normoxia (N, 21% O_2) and hypoxia (H, 0.5% O_2) and incorporation into glucose-6-phosphate (glucose-6P) and redistribution of ^{13}C carbons was measured using LC-MS. Data represents mean ±SEM (n=3).

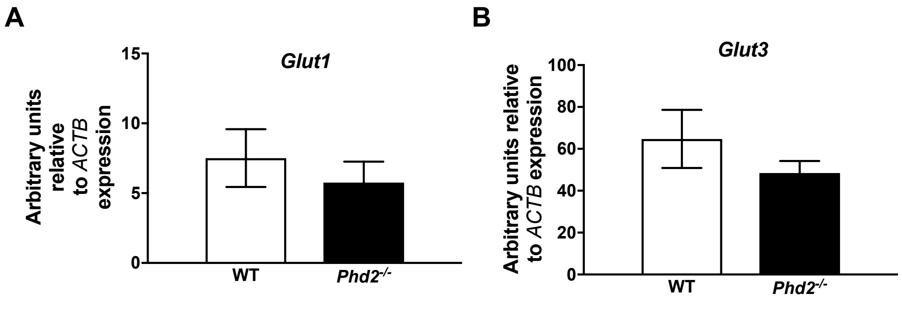

Supplementary figure 6. Wild-type and *Phd2*$^{-/-}$ bone marrow neutrophils display equivalent expression of glucose transporters at the mRNA level

Bone marrow neutrophils of LPS nebulised wild-type and *Phd2*-deficient mice were lysed and TaqMan analysis of cDNA performed with data normalised to β-*Actin* expression. Data represent mean ±SEM, n=4.
